# Fate and Complex Pathogenic Effects of Dioxins and Polychlorinated Biphenyls in Obese Subjects before and after Drastic Weight Loss

**DOI:** 10.1289/ehp.1002848

**Published:** 2010-12-15

**Authors:** Min-Ji Kim, Philippe Marchand, Corneliu Henegar, Jean-Philippe Antignac, Rohia Alili, Christine Poitou, Jean-Luc Bouillot, Arnaud Basdevant, Bruno Le Bizec, Robert Barouki, Karine Clément

**Affiliations:** 1 INSERM, UMR-S 747, Université Paris Descartes, Centre Universitaire des Saints-Pères, Paris, France; 2 Ecole Nationale Vétérinaire, Agroalimentaire et de l’Alimentation Nantes Atlantique (ONIRIS), Laboratoire d’Etude des Résidus et Contaminants dans les Aliments (LABERCA), INRA USC 2013, Nantes, France; 3 INSERM, U872, Nutriomique team 7, Centre de Recherche des Cordeliers, Université Pierre et Marie Curie-Paris 6, UMR S 872, Paris, France; 4 Assistance Publique-Hôpitaux de Paris, Pitié-Salpêtrière Hospital, Nutrition and Endocrinology Department, Paris, France; 5 Centre de Recherche en Nutrition Humaine-Ile de France, Paris, France; 6 Assistance Publique-Hôpitaux de Paris, Hôtel-Dieu Hospital, Surgery Department, Paris, France; 7 Assistance Publique-Hôpitaux de Paris, Hôpital Necker-Enfants Malades, Paris, France

**Keywords:** adipose tissue, bariatric surgery, dioxin, obesity, PCB, persistent organic pollutants, xenobiotic metabolizing enzymes

## Abstract

**Background:**

In humans, persistent organic pollutants (POPs) are stored primarily in adipose tissue. Their total body burden and their contribution to obesity-associated diseases remain unclear.

**Objectives:**

We characterized POP total body burden and their redistribution in obese individuals before and after drastic weight loss and compared these values with a variety of molecular, biological, and clinical parameters.

**Methods:**

Seventy-one obese subjects were enrolled and underwent bariatric surgery. Blood and adipose tissue samples were obtained at different times from these individuals as well as from 18 lean women.

**Results:**

POP content (17 dioxins/furans and 18 polychlorinated biphenyl congeners) in different adipose tissue territories was similar, allowing us to assess total POP body burden from a single biopsy. Total POP body burden was 2 to 3 times higher in obese than in lean individuals. We also found increased expression of some POP target genes in obese adipose tissue. Drastic weight loss led to increased serum POPs and, within 6–12 months, to a significant 15% decrease in total polychlorinated biphenyl body burden. Importantly, serum POP levels were positively correlated with liver toxicity markers and lipid parameters, independently of age and body mass index.

**Conclusions:**

POP content in adipose tissue and serum correlate with biological markers of obesity-related dysfunctions. Drastic weight loss leads to a redistribution of POPs and to a moderate decrease of their total body burden.

Persistent organic pollutants (POPs) are under scrutiny because their sustainable storage in the adipose tissue might lead to a chronic internal exposure, possibly disrupting metabolic and endocrine functions. Recently, human studies have provided indirect evidence for the possible implication of these environmental pollutants in metabolic disorders. Few studies have reported correlations between the plasma concentrations of certain POPs and metabolic traits (Ha et al. 2007, 2009; Lee et al. 2008; Uemura et al. 2009). These epidemiologic studies are in line with *in vitro* and animal data suggesting that POPs such as dioxin could impair insulin secretion and action (Liu and Matsumura 2006; Marchand et al. 2005; Piaggi et al. 2007) and interfere with adipogenesis (Liu et al. 1998; Shimba et al. 2001) and adipose tissue function (Brewster and Matsumura 1988; Lakshman et al. 1988). However, POPs comprise a large variety of pollutant families. Although these chemical families share physicochemical properties, their biological effects are diverse. Whereas polychlorinated dibenzo-*p*-dioxin (PCDDs), polychlorinated dibenzofurans (PCDFs), and “dioxin-like” polychlorinated biphenyls (dl-PCBs) act primarily through the activation of the aryl hydrocarbon receptor (AhR), other PCBs, brominated flame retardants, and organochlorine pesticides exhibit different modes of action. Within a single family of pollutants such as dioxins, congeners exhibit different potencies, and little is known about their kinetic behavior in the organism, their distribution, or their bioavailability (Van den Berg et al. 2006).

Characterizing dynamic conditions leading to the release and redistribution of these pollutants can provide insights into the profile and kinetics of POPs and their putative pathogenic effects in humans. Tremblay’s group in Canada studied the variations of plasma concentrations of selected POPs such as PCBs and organochlorine pesticides after diet-induced weight loss (Chevrier et al. 2000; Imbeault et al. 2002). Moderate weight loss was associated with increased plasma concentrations of these POPs, suggesting a release of pollutants from fat mass that could be related to increased lipolysis. These effects were also observed during weight loss induced by bariatric surgery (Charlier et al. 2002; Hue et al. 2006). These initial studies suggest that surgical or diet-induced weight loss represents a valuable model to study the effects of body POP redistribution on clinical and biological parameters in humans.

Critical questions remained unanswered. First, because body fat constitutes the reservoir of POPs, it is important to assess their adipose tissue concentrations, as well as their total body burden, in addition to their plasma levels in order to evaluate their kinetic behavior. Furthermore, although visceral and subcutaneous fat territories display different metabolic properties, it is unclear whether they behave similarly in terms of POP storage. Second, a detailed characterization of all congeners within a family of pollutants is required to determine whether one of them could be representative of the others. Third, in addition to POP kinetics, it is important to assess their biological effects, for example, by studying their target gene expression, and to evaluate relationships between changes in POP abundance and various bioclinical variations occurring after body fat loss.

For this purpose, we measured a wide range of dioxins, furans, and PCB congeners in different fat depots and plasma samples collected from lean and obese individuals and assessed total body burden before and after weight loss. We followed obese patients undergoing weight loss through bariatric surgery and compared the POP profile, gene expression in adipose tissue depots and in blood, and a variety of biological and clinical parameters.

## Materials and Methods

### Subjects

A total of 71 obese subjects involved in a gastric surgery program were recruited between 2006 and 2008 in the Department of Nutrition, Center of Reference for Medical and Surgical Care of Obesity, Pitié-Salpêtrière Hospital (Paris, France). The ethics committee of the Hôtel-Dieu Hospital approved the clinical investigations both for obese and nonobese individuals. All subjects gave a written informed consent after individual explanation. Patients met the criteria for obesity surgery [body mass index (BMI) ≥ 40 kg/m^2^, or ≥ 35 kg/m^2^ with at least one comorbidity (hypertension, type 2 diabetes, dyslipidemia, or obstructive sleep apnea syndrome); for the scheme of the study design, see Supplemental Material, Figure 1 (doi:10.1289/ehp.1002848)].

### Clinical and biological parameters

Adiposity-related markers were determined. BMI (kilograms per square meter) was calculated from the measured body weight with the same scale and height. Fat and fat-free mass were determined by dual-energy X-ray absorptiometry (DXA; GE Lunar Prodigy Corp., Madison, WI, USA) (Table 1). Resting energy expenditure was measured in the obese subjects by indirect calorimetry after 12 hr of fasting (Quark Resting Metabolic Rate; Delta Medical, Soucieu-en-Jarrest, France). These evaluations were performed before and during the postsurgery follow-up. Periumbilical surgical needle biopsies of subcutaneous adipose tissue were obtained before Roux-en-Y gastric bypass (RYGB) and during the follow-up, and omental adipose tissue was also collected at the time of surgery, as previously described (Mutch et al. 2009). After adipocyte isolation and collagenase digestion, adipocyte diameter was measured as described elsewhere (Prat-Larquemin et al. 2004).

Blood samples were obtained at each time point in all subjects after 12 hr of fasting and stored at −20°C for later analysis. Biological parameters included lipid [total cholesterol, high-density lipoprotein (HDL) cholesterol, and triglycerides], insulin and glucose values, leptin, adiponectin, and inflammatory markers [e.g., highly sensitive C-reactive protein (hsCRP), interleukin (IL)-6, orosomucoid]. Liver markers [aspartate transaminase (AST), alanine transaminase (ALT), and γ-glutamyltransferase (γGT)] were measured by routine tests. Homeostatic model assessment (HOMA) for insulin sensitivity (HOMA-S) and beta-cell function (HOMA-B) were estimated using the method described by Matthews et al. (1985). Metabolic syndrome traits in obese and lean subjects were clinically assessed based on consensual criteria proposed by Alberti et al. (2009).

### PCDD/PCDF and PCB analysis

All organic solvents (Promochem, Molsheim, France) were picograde quality. Silica (Fluka, Buchs, Switzerland), sodium sulfate and dipotassium oxalate (Merck, Darmstad, Germany), and acetic acid and sodium dodecyl sulfate were of superior analytical quality. Native and ^13^C-labeled standards were purchased from Cambridge Isotope Laboratories (Andover, MA, USA) and Wellington Laboratory (Guelph, ON, Canada). Standard solutions were prepared in toluene or isooctane for (PCDD/PCDFs) and PCBs, respectively. All reference solutions were stored in darkness at < 6°C.

Before extraction, 17 ^13^C-labeled PCDD/PCDFs and 18 ^13^C-labeled PCBs congeners were added to each sample for internal standard calibration and quantification by the isotope dilution method. Sample preparation is described in detail in Supplemental Material (doi:10.1289/ehp.1002848).

PCDD/PCDF and PCB measurements were performed by gas chromatography coupled to high-resolution mass spectrometry using an HP-5890 gas chromatograph (Hewlett Packard, Palo Alto, CA, USA) coupled to a JMS 700D or a JMS 800D double electromagnetic sector high resolution mass spectrometer (Jeol, Tokyo, Japan). Analytical methods are described in detail in Supplemental Material (doi:10.1289/ehp.1002848).

Total POP burden is calculated by taking into account the concentration of POPs per gram of lipid in the adipose tissue and the total amount of fat in the body as calculated by DXA. Because, under the conditions of the study, POPs are primarily associated with fat, this value is a good estimate of total body burden.

### Determination of target gene expression

Total RNA was extracted from adipose tissue with the RNeasy total RNA Mini kit (Qiagen, Courtaboeuf, France). Total RNA concentration was assessed spectrophotometrically with a NanoDrop (Labtec, Palaiseau, France) and their quality was determined with the Agilent 2100 Bioanalyzer (Agilent Technologies, Massy, France). Total RNA (1 μg) was reverse transcribed using a high-capacity cDNA reverse transcription kit (Applied Biosystems, Courtaboeuf, France). Real-time polymerase chain reactions (PCRs) were conducted with absolute QPCR SYBR Green Rox mix (Thermo Electron SAS, Villebon sur Yvette, France) on ABI Prism 7900 (Applied Biosystems, Courtaboeuf, France). mRNA values were normalized to the expression level of a reference gene by using the DDCT method (comparative Ct method) described in Supplemental Material (doi:10.1289/ehp.1002848). Primer sequences are available upon request.

### Statistical analysis

Quantitative variables, including clinical and biological parameters as well as various POP contamination levels, measured in serum and adipose tissue samples are expressed as mean ± SE. We used Wilcoxon rank sum tests to assess statistical significance of differences in clinical and biological parameters and in POP contamination levels, between lean and obese subjects at baseline. Paired Wilcoxon rank sum tests were performed to analyze changes in these parameters between various time points after surgery. Multivariate analysis of variance was used to explore global variations of the analyzed parameters throughout the follow-up after surgery.

We used principal component analysis (PCA) combined with co-inertia analysis to explore complex and potentially redundant relationships involving a relatively large number of clinical and biological parameters, as well as POP contamination levels, at baseline and after RYGB. Co-inertia analysis is a coupling method for comparing different types of parameters presenting different variances. To estimate correlation between POPs and bioclinical variable, we used partial Spearman correlation coefficients (p*R*_s_) estimated by considering potentially confounding factors, such as age and sex, in a variance-covariance matrix computation.

The significance of the strongest dynamic associations, among those identified by PCA and co-inertia analysis, involving clinical-biological parameters and POP contamination levels, was further evaluated by building linear mixed-effects (LME) models to test for intervariable redundancies and to adjust for potential confounding factors. Age and sex were systematically considered as confounding covariates in LME models. All LME models were fit by maximizing the restricted log-likelihood of their estimated coefficients. Statistical analyses were performed using the R software (R Project for Statistical Computing, Vienna, Austria; http://www.r-project.org). PCA and co-inertia analyses were performed with “ade4” R package. LME modeling was performed by relying on statistical functions available in the “nlme” R package. All statistical computations with *p*-values < 0.05 were considered significant.

## Results

### Clinical and biological characteristics before and after weight loss

Table 1 presents clinical characteristics of lean and obese subjects. As expected, obese subjects had higher leptin, fasting glucose, insulin, triglyceride serum, and inflammatory marker concentrations and lower adiponectin and HDL cholesterol serum concentrations compared with the lean ones.

Table 2 presents the profile of clinical and biological parameters related to body composition, metabolic, inflammatory, and liver markers in obese subjects. The amount of fat mass, adipocyte cell diameter, and serum leptin concentrations decreased significantly, as expected. Bioclinical modifications were in agreement with an improvement of biological, metabolic, liver, and inflammatory parameters after RYGB.

### POP levels in blood and different adipose depots

**Figure 1:**
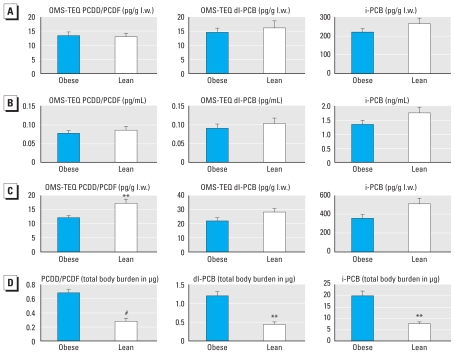
Serum and adipose tissue POPs in lean and obese subjects. POPs were measured in serum of 35 obese patients before surgery and in 17 lean subjects. (*A,B*) Measured POPs expressed on a lipid weight (l.w.) basis (*A*) or expressed on fresh weight basis (*B*) did not differ. Wilcoxon rank sum test was used for comparison. (*C*) Concentrations of POPs measured in adipose tissue of 18 lean versus 65 obese patients (before surgery). (*D*) Total body burdens (total charge) of POPs measured in adipose tissue of 12 lean versus 65 obese patients (before surgery), relative to the total fat mass. Twelve lean subjects and all obese subjects agreed to the DXA scan. Wilcoxon rank sum test was used for comparison. **p < 0.01. ^#^*p* < 0.001.

We examined the distribution of each pollutant congener, both in blood of lean versus obese subjects and in two adipose tissue depots in obese subjects. We next calculated the POP contamination level for each class of pollutants. The POP contamination levels were statistically similar in serum from obese versus lean patients, either on a lipid weight basis (picograms or nanograms per lipid weight) or on a fresh weight basis (picograms or nanograms per milliliter) (Figure 1A,B). Results obtained for adipose tissue are presented in Figure 1C, showing a significantly higher concentration (classically expressed relative to the extracted fat content, i.e., on a lipid weight basis) of PCDD/Fs in lean subjects compared with obese subjects, whereas this tendency was not statistically significant for dl-PCBs and was borderline for indicator PCBs (i-PCBs); indicator PCBs (also known as marker PCBs) include PCBs 28, 52, 101, 118, 138, 153 and 180.

We next compared different adipose tissue territories. In a subset of obese subjects for which paired samples were available (*n* = 7), we found a strong correlation between the measured contamination levels in visceral (omental tissue) and subcutaneous depots before surgery for PCDD/PCDFs (*R*_s_ = 0.98, *p* < 0.0001), dl-PCBs (*R*_s_ = 0.99, *p* < 0.0001), or i-PCBs (*R*_s_ = 0.99, *p* < 0.0001). We therefore concluded that the easily accessible subcutaneous depot pollutant concentrations represent a reliable estimate of adipose tissue contamination by pollutants.

To obtain a more global and realistic estimation of POP contamination, and because POP concentration did not seem to be influenced by the adipose tissue territory, we calculated the total body burden of pollutants in each individual by multiplying the concentration values (expressed on a lipid weight basis) by the total fat mass estimated by DXA (“total POP burden”). The total POP burden in adipose tissue was increased by 2.7-, 2.9-, and 2.8-fold in obese versus lean patients for PCDD/PCDFs (*p* < 0.0001), dl-PCBs (*p* < 0.001), and i-PCBs (*p* < 0.001) (Figure 1D). In obese subjects for which the two type of samples were available (*n* = 32), we observed a significant and strong correlation before surgery between the contamination levels in adipose tissue and blood for PCDD/PCDFs (*R*_s_ = 0.87, *p* < 0.0001), i-PCBs (*R*_s_ = 0.96, *p* < 0.0001), or dl-PCBs (*R*_s_ = 0.92, *p* < 0.0001). In lean subjects (*n* = 13), we found weaker correlations with *R*_s_ equal to 0.61 (*p* < 0.05), 0.80 (*p* < 0.001), and 0.35 (*p* < 0.05), respectively, for PCDD/PCDFs, i-PCBs, and dl-PCBs [see Supplemental Material, Figure 2 (doi:10.1289/ehp.1002848)]. We observed strong positive correlations between subjects’ age and POP contamination levels measured in either adipose tissue (see Supplemental Material, Figure 3) or blood. Again, these correlations with age were stronger in obese than in lean subjects (*R*_s_ = 0.73 vs. 0.31, 0.68 vs. 0.41, and 0.52 vs. 0.52 for PCDD/PCDFs, dl-PCBs, and i-PCBs, respectively).

### Expression of pollutant target genes

**Figure 2:**
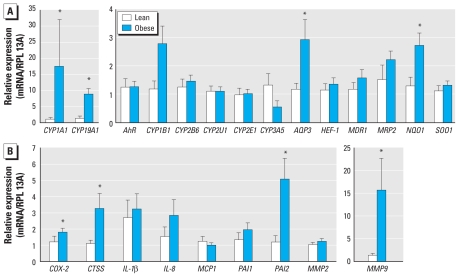
Expression of pollutant target genes in adipose tissue of lean and obese subjects. High-quality RNA was available for seven lean and nine obese subjects. The relative expression was obtained by RTqPCR using the comparative Ct method by reporting Ct of target gene of each subject to mean Ct of lean subjects and by using *RPL13A* (60S ribosomal protein L13a) as reference gene. (*A*) *AhR*, phase I xenobiotic metabolizing CYP genes and other targets of the xenobiotic receptors. (*B*) Targets of the xenobiotic receptors involved in obesity-associated low-grade inflammation. For each gene, data for obese and lean subjects were statistically compared by Wilcoxon rank sum test. *p < 0.05.

We examined by reverse-transcriptase quantitative polymerase chain reaction (RTqPCR) the expression of a series of adipose tissue genes known to be induced by these pollutants as well as that of other relevant genes in subgroups of control and obese individuals, including only females. Several typical targets of the AhR, such as *CYP1A1* (cytochrome P450 1A1), *CYP19A1*, and *NQO1* [NAD(P)H,quinone oxidoreductase-1], were up-regulated in the adipose tissue of obese individuals (Figure 2). Interestingly, the expression of genes involved in low-grade inflammation associated with obesity such as *COX-2* (cyclooxygenase 2), *CTSS* (cathepsin S), and *MMP9* (matrix metalloproteinase-9), which are also AhR target genes, was elevated in obese patients. These observations suggest that obesity is accompanied by an increased expression of POP-target genes. However, other obesity-related factors may account for these observations. We also monitored gene expression in blood cells, but this gave less conclusive data. Among POP target genes, only *CYP1A1* and *IL1b* (interleukin-1β) gene expression was significantly increased, independent of sex or age [see Supplemental Material, Figure 4 (doi:10.1289/ehp.1002848)].

### Association of POP levels with metabolic and liver parameters

PCDD/PCDF concentrations in adipose tissue (expressed per gram of lipids) correlated negatively both with body weight (p*R*_s_ = −0.31, *p* < 0.05) and BMI (p*R*_s_ = −0.25, *p* < 0.05), independent of age. We also found similar associations with other PCBs [see Supplemental Material, Table 1 (doi:10.1289/ehp.1002848)].

We also observed significant positive associations between POP contamination levels in blood (picograms or nanograms per liter) and a series of liver and metabolic parameters, meaning that the higher the POP concentration in blood, the more deteriorated the liver function in these obese subjects. For example, serum i-PCBs correlated with AST (p*R*_s_ = 0.64, *p* < 0.0001), ALT (p*R*_s_ = 0.40, *p* < 0.05), and γGT (p*R*_s_ = 0.58, *p* < 0.001). We also observed positive correlations with blood lipids as serum level of triglycerides (p*R*_s_= 0.36, *p* < 0.0001 for i-PCBs) and total cholesterol (p*R*_s_ = 0.38, *p* < 0.05 for i-PCBs). Data for other pollutants are shown in Supplemental Material, Table 1 (doi:10.1289/ehp.1002848).

### POP levels and gene expression during weight loss

**Figure 3:**
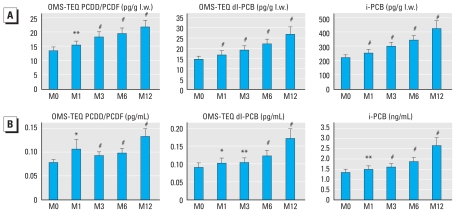
Serum POPs concentrations before and after bariatric surgery in obese subjects: time course of the concentrations of POPs measured in serum of 37 obese patients before [month 0 (M0)] and 1, 3, 6, and 12 months after surgery expressed on a lipid weight (l.w.) basis (*A*) or on a fresh weight basis (*B*). At M12, there was an increase of 38%, 45%, and 48%, respectively, for PCDD/Fs, dl-PCBs, and i-PCBs versus M0. *p < 0.05, **p < 0.01, ^#^*p* < 0.001, Wilcoxon rank sum test comparing M1, M3, M6, and M12 versus M0.

We examined changes in pollutant abundance both in serum and adipose tissue during surgery-induced weight loss. Surgery-induced weight loss was associated with a gradual and significant increase in the serum concentrations of POPs measured in obese patients whether expressed on a lipid weight basis or per milliliter of serum (Figure 3). At 12 months, the mean serum concentrations of pollutants were increased by 38%, 46%, and 48% compared with basal levels for PCDD/PCDFs, dl-PCBs, and i-PCBs respectively (Figure 3). The effect of this drastic perturbation on pollutant distribution was similar for all congeners.

**Figure 4:**
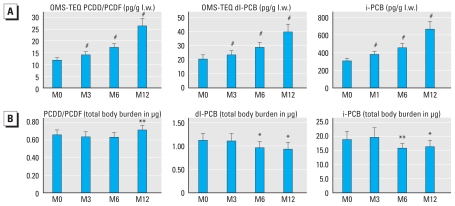
Adipose tissue POP concentrations before and after bariatric surgery in obese subjects. (*A*) Time course of the concentrations of POPs measured in adipose tissue of obese patients before and after surgery expressed on a lipid weight (l.w.) basis. Measures were obtained in 37 obese subjects. At 12 months (M12), there was an increased concentration of 54%, 45%, and 64%, respectively, for PCDD/Fs, dl-PCBs, and i-PCBs versus before surgery (M0). (*B*) Time course of the amounts (total charge) of total body burdens of POPs measured in adipose tissue of obese patients before and after surgery (expressed in picograms and deduced for each POP family from the concentration reported in A and the total fat mass). Measures were obtained in 37 obese subjects. *p < 0.05, **p < 0.01, ^#^*p* < 0.001 (Wilcoxon rank sum test comparing M3, M6, and M12 vs. M0).

We observed a gradual increase in the concentrations of POPs (i.e., picograms or nanograms per gram lipid weight) in subcutaneous adipose tissue of obese subjects after weight loss (Figure 4A). In contrast, the total body burden of POPs, as calculated from adipose tissue concentration and total fat mass, decreased continuously for dl-PCBs (*p* < 0.05) and for i-PCBs (*p* < 0.05) (Figure 4B). The decrease was approximately 10–15% at 6 and 12 months postsurgery. After an initial decrease at 3 and 6 months, PCDD/F total body burden increased slightly but significantly at 12 months after versus before surgery (*p* < 0.01) (Figure 4B).

We evaluated gene expression in adipose tissue of obese subjects before and 6 months after surgery. Although some AhR target genes such as *NQO1*, *SOS1* (son of sevenless homolog 1), and *AQP3* (aquaporin 3) decreased 6 months after surgery, *CYP1B1* and *CYP1A1* did not vary significantly. Other xenobiotic metabolizing enzyme genes such as *CYP2B6*, *CYP2U1* were significantly decreased after 6 months of weight loss. The decrease in inflammatory genes such as *COX-2* and *MMP9* also known to be POP target genes indicates that the expression of several POP target genes in adipose tissue decreased after weight loss, whereas that of other targets was either unchanged or slightly increased [see Supplemental Material, Figure 5A (doi:10.1289/ehp.1002848)].

We also monitored gene expression in blood at 0, 1, 3, 6, and 12 months. Although the expression of several inflammatory genes decreased significantly over time, the expression of direct AhR target genes did not vary significantly, although we observed a clear tendency for a decrease in the expression of the *CYP1A1* and *CYP1B1* genes [see Supplemental Material, Figure 5B (doi:10.1289/ehp.1002848)].

### Dynamic association studies during weight loss

**Figure 5:**
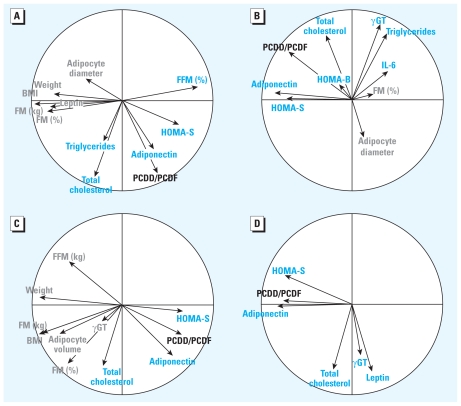
Associations among variations of POPs serum and adipose tissue levels and bioclinical parameters: correlation circles computed through PCA illustrating significant associations among variations of POP levels in serum (*A* and *B*) or adipose tissue (*C* and *D*) (indicated here for PCDD/PCDFs) before (*A*,*C*) and after (*B*,*D*) adjusting for variations in total body fat mass after RYGB in an LME model. Positive associations with PCDD/PCDF are indicated in blue, and negative ones in gray (FFM, fat-free mass; FM, fat mass). Similar results were obtained for the other two congeners [Supplemental Material, Tables 2, 3 (doi:10.1289/ehp.1002848)].

Variations of all POP contamination levels after RYGB, in blood and adipose tissue, displayed strong negative associations with a series of corpulence-related parameters [Figure 5; see also Supplemental Material, Table 2 (doi:10.1289/ehp.1002848)], including BMI, body fat mass, leptin levels, and adipocyte volume. Interestingly, some of these associations remained significant even after adjustment for body fat mass variations (see Supplemental Material, Tables 2,3). For example, PCDD/PCDF blood levels showed negative correlations with BMI (p*R*_s_ = −0.44, *p* < 0.00001), adipocyte diameter (p*R*_s_ = −0.27, *p* < 0.01), and leptin serum levels (p*R*_s_ = −0.29, *p* < 0.05) and a positive correlation with adiponectin serum levels (p*R*_s_ = 0.30, *p* < 0.01). Some of these relationships remained significant after adjusting for body fat mass variations, including those with adipocyte diameter (p*R*_s_ = −0.13, *p* < 0.01) and adiponectin serum levels (p*R*_s_ = 0.23, *p* < 0.01). We also found a negative correlation between resting metabolic rate and change in blood pollutants (for PCDD/PCDFs, p*R*_s_ = −0.27, *p* < 0.01). The association depended on the changes of body composition–related parameters, such as total lean and fat mass or BMI, because the association disappeared after adjustment on these factors (data not shown).

Variations in POP contamination levels after surgery also displayed strong positive associations with metabolic and hepatic parameters [see Supplemental Material, Table 2, Figure 5 (doi:10.1289/ehp. 1002848)]. Importantly, most of these associations were independent of the variations in body fat mass after surgery, thus indicating that the increase in POP concentrations after surgery associates with a delayed normalization of lipid- and liver-related parameters. For example, PCDD/PCDF blood concentrations correlated positively with serum levels of triglycerides (p*R*_s_ = 0.20, *p* < 0.01), total cholesterol (p*R*_s_ = 0.40, *p* < 0.00001), and γGT (p*R*_s_ = 0.14, *p* < 0.01), independent of body fat mass variations. Surprisingly, we observed a positive association between concentrations of various POPs in adipose tissue and in blood and the improvement of HOMA-S (indicator of insulin sensitivity) and HOMA-B (indicator of pancreatic insulin secretion) after surgery even after adjustment for body fat mass variations.

## Discussion

In the present study, we carried out a novel and comprehensive assessment in humans of the distribution and putative effects of several POPs that are known to be preferentially localized into adipose tissue. We conducted the study in obese individuals undergoing drastic weight loss, a unique condition leading to the redistribution of endogenous POPs. We first showed that the patterns of POP congeners distributions in the subcutaneous and visceral adipose tissue depots are extremely similar. This has already been shown for some organochlorine pollutants in postmortem studies by Dewailly et al. (1999). This observation has important practical implications because it suggests that the easily accessible subcutaneous adipose tissue is representative of the pollutants distribution in deeper fat depots and allowed us to estimate total POP body burden.

An important observation of this study was that the POP body burden is significantly higher in obese than in lean individuals, confirming and extending some previous studies (Wolff et al. 2005). However, the concentrations of adipose tissue POPs (per gram of lipid) in obese individuals were either smaller or similar to those of lean subjects, indicating that POPs are more dilute in obese fat mass and that the increased body burden is due to the larger fat mass compartment. We also observed increased POP target gene expression in the adipose tissue of obese individuals, but the interpretation of such an increase is unclear at this stage.

The study also highlights the importance of the size of the triglyceride droplet (i.e., mostly conditioning the adipocyte size) in controlling the release of POPs into the bloodstream, because diminished adipocyte size and weight loss have an impact on POP serum level. These data suggest that increased lipolysis associated with weight loss may lead to a faster exchange of POPs between the different body compartments. Nevertheless, we show that drastic weight loss leads to a significant 10–15% decrease of the body burden of the most abundant POPs (i.e., dl-PCBs and i-PCBs) but not for PCDD/PCDFs. Although there is no clear explanation for the latter observation, POP body burden tends naturally to increase, at a rate of approximately 3–5% per year [see Supplemental Material, Figure 3 (doi:10.1289/ehp.1002848)]. In addition, it is unclear whether the decrease in PCB body burden is due to elimination, as we suspect, or to a redistribution in a different nonfat compartment.

We addressed the impact of POP redistribution on metabolism by exploring basal and dynamic correlations between POP release and the subjects’ metabolic changes. In agreement with previous studies on organochlorine chemicals (reviewed by Pelletier et al. 2003), we observed a significant negative association between serum POPs and resting metabolic rate that is known to diminish after weight loss. However, this association did not remain when we adjusted comparisons for corpulence-related parameters. This result indicates that POP release from adipose tissue and variations in body corpulence are not independent factors for the control of resting metabolic rates, suggesting that increased serum POPs may at least partially account for the decreased energy expenditure elicited by weight loss. Data from the literature suggest that such effects could be mediated by alteration in thyroid hormone effects or metabolism (Pelletier et al. 2003). We also observed a positive relationship between plasma POPs and lipid (i.e., triglycerides and cholesterol) and liver (i.e., transaminases and γGT) parameters. In addition, although it is widely accepted that weight loss associates with metabolic and liver function improvements (Mathurin et al. 2009), we here observed a positive association between a diminished improvement of lipid values and of liver markers and increased POP plasma concentrations. Thus, although the beneficial effects of weight loss are always observed, they appear to be slowed down or decreased by higher POP levels. Obesity is a well-known important risk factor for nonalcoholic fatty liver disease, cirrhosis, and cancer (Siegel and Zhu 2009). The pathologic transformations of the adipose tissue in obese subjects, particularly increased cytokine and free fatty acid release, contribute to nonalcoholic fatty liver disease physiopathology (Brunt 2010; Tiniakos et al. 2010). Thus, our study raises the issue of a possible contribution to liver dysfunction of chronic internal exposure of obese subjects to POPs, in addition to other factors.

The assumption that POPs may jeopardize all aspects of obese individual health is not straightforward. This is illustrated in our study, showing a positive correlation between the increase of plasma POPs and the improvement of insulin sensitivity even after adjustment for body fat mass. Although at first view these findings appear surprising based on previous observations suggesting an association between POPs and the incidence of diabetes (Fierens et al. 2003; Lee et al. 2008), they are in agreement with a previous exploration in less obese men showing that the increase of organochlorine pollutants in human serum was positively associated with the decrease in insulin levels after diet-induced weight loss (Imbeault et al. 2002). One possible mechanistic explanation for these paradoxical effects can be deduced from studies showing that POPs such as dioxin lead to a decrease in liver gluconeogenesis in several cellular and animal models mainly through the repression of phosphenolpyruvate carboxykinase (Viluksela et al. 1999). Thus decreased liver glucose production may compensate other deleterious metabolic effects of pollutants and lead to improved insulin-sensitivity biomarkers.

In conclusion, the comparison of obese and lean individuals, as well as the follow-up of obese subjects during weight loss, indicates that, in humans, POPs could be involved in the alteration of energy and lipid homeostasis and in liver dysfunction.

## Figures and Tables

**Table 1:** Characteristics of obese subjects before surgery and a group of normal-weight subjects.

Clinical and biological parameters	Obese subjects (*n* = 71)	Lean subjects (*n* = 18)	*p*-Value^a^
Age (years)	44 ± 1.6	39.18 ± 2.85	NS

Adiposity markers			

BMI (kg/m^2^)	48 ± 0.79	21.67 ± 0.36	< 0.0001
Body weight (kg)	130.58 ± 2.98	59.1 ± 1.56	< 0.0001
Fat mass (kg)	58.21 ± 1.46	15.7 ± 9.7^b^	< 0.0001
Fat-free mass (kg)	62.84 ± 1.46	40.6 ± 11^b^	< 0.0001
Leptin (ng/mL)	54.95 ± 3.27	8.83 ± 1.11	< 0.001

Plasma glucose homeostasis			

Glycemia (mmol/L)	6.07 ± 0.26	4.38 ± 0.12	< 0.01
Insulinemia (μU/mL)	18.27 ± 1.43	3.83 ± 0.38	< 0.0001
Adiponectin (μg/mL)	8.26 ± 0.93	13.39 ± 1.14	< 0.01

Plasma lipid homeostasis			

Total cholesterol (mmol/L)	4.76 ± 0.12	5.02 ± 0.27	NS
Total triglycerides (mmol/L)	1.36 ± 0.08	0.89 ± 0.08	< 0.0001
HDL cholesterol (mmol/L)	1.26 ± 0.04	1.69 ± 0.09	< 0.0001

Inflammatory markers			

Plasma hsCRP (mg/L)	10.48 ± 1.12	3.43 ± 1.02	< 0.0001
Plasma IL6 (pg/mL)	9.12 ± 2.26	3.35 ± 1.68	< 0.0001

NS, not significant.

a Wilcoxon rank sum tests were used to compare obese and lean subjects.

b Data available in 12 subjects who agreed to the DXA measure.

**Table 2:** Clinical and biological characteristics of subjects before and 3, 6, and 12 months after surgery.

		After bypass			
Clinical and biological parameters	Before bypass	3 months	6 months	12 months	*p*-Value^a^
Adiposity markers					

Body weight (kg)	130 ± 2.98	110.06 ± 2.60	99.93 ± 2.32	93.01 ± 2.48	< 0.0001
Resting energy expenditure (Kcal/24 hr)	1955.3 ± 49.8	NA	1604.7 ± 34.52	1582.4 ± 42.12	< 0.0001
BMI (kg/m^2^)	47.77 ± 0.79	40.28 ± 0.73	37.61 ± 0.70	34.03 ± 0.72	< 0.0001
Adipocyte diameter (μm)	115.46 ± 2.14	110.14 ± 2.24	105.53 ± 1.20	97.88 ± 1.73	< 0.0001
Fat mass (kg)	58.21 ± 1.46	48.26 ± 1.42	41.01 ± 1.31	33.31 ± 1.48	< 0.0001
Fat-free mass (kg)	62.84 ± 1.46	57.83 ± 1.46	55.57 ± 1.37	55.57 ± 1.61	< 0.0001
Leptin (ng/mL)	54.95 ± 3.27	26.69 ± 1.59	21.09 ± 1.54	17.61 ± 1.82	< 0.0001

Plasma glucose homeostasis and insulin sensitivity parameters					

Glycemia (mmol/L)	6.07 ± 0.26	4.97 ± 1.11	4.79 ± 0.10	4.73 ± 0.15	< 0.0001
Insulinemia (μU/mL)	18.27 ± 1.43	9.00 ± 1.64	6.71 ± 0.56	6.33 ± 0.59	< 0.0001
HOMA-B	129.70 ± 6.71	108.86 ± 5.65	92.02 ± 5.34	102.94 ± 12.24	< 0.01
HOMA-S	61.28 ± 4.67	116.33 ± 8.19	175.21 ± 15.84	194.23 ± 22.91	< 0.0001
Adiponectin (μg/mL)	8.26 ± 0.93	8.11 ± 0.59	8.52 ± 0.54	11.03 ± 0.87	< 0.001

Plasma lipid homeostasis					

Total cholesterol (mmol/L)	4.76 ± 0.12	4.34 ± 0.09	4.21 ± 0.12	4.37 ± 0.13	< 0.01
Total triglycerides (mmol/L)	1.36 ± 0.08	1.19 ± 0.05	1.12 ± 0.06	0.94 ± 0.007	< 0.0001
HDL cholesterol (mmol/L)	1.26 ± 0.04	1.17 ± 0.03	1.29 ± 0.04	1.74 ± 0.28	< 0.01

Liver tests					

AST	27.97 ± 1.61	29.84 ± 1.31	25.90 ± 1.14	26.98 ± 1.67	< 0.05
ALT	38.65 ± 4.48	36.30 ± 3.01	28.86 ± 2.69	28.83 ± 2.65	< 0.05
γGT	52.97 ± 8.97	30.61 ± 4.41	26.39 ± 3.32	30.76 ± 5.16	< 0.001

Inflammatory markers					

Plasma hsCRP (mg/L)	10.48 ± 1.12	5.64 ± 0.74	4.29 ± 0.62	2.71 ± 0.61	< 0.0001
Plasma IL6 (pg/mL)	9.12 ± 2.26	4.05 ± 0.56	3.41 ± 0.48	2.01 ± 0.22	0.0852

a Time-dependent changes were evaluated by multivariate analysis of variance.
